# Plant Responses to Hypoxia: Signaling and Adaptation

**DOI:** 10.3390/plants9121704

**Published:** 2020-12-03

**Authors:** Elena Loreti, Gustavo G. Striker

**Affiliations:** 1 Institute of Agricultural Biology and Biotechnology, CNR, National Research Council, Via Moruzzi, 56124 Pisa, Italy; 2 IFEVA, CONICET, Cátedra de Fisiología Vegetal, Facultad de Agronomía, Universidad de Buenos Aires, Av. San Martín 4453, Buenos Aires C1417DSE, Argentina

## 1. Introduction

Molecular oxygen deficiency leads to altered cellular metabolism and can dramatically reduce crop productivity. Nearly all crops are negatively affected by lack of oxygen (hypoxia) due to adverse environmental conditions such as excessive rain and soil waterlogging. Extensive efforts to fully understand how plants sense oxygen deficiency and their ability to respond using different strategies are crucial to increase hypoxia tolerance. It was estimated that 57% of crop losses are due to floods [1]. Progress in our understanding has been significant in the last years. This topic deserved more attention from the academic community; therefore, we have compiled a Special Issue including four reviews and thirteen research articles reflecting the advancements made thus far.

## 2. Advances in Hypoxia Sensing and Responses

*Oryza sativa* (rice) is an important crop widely used in areas prone to suffer waterlogging and submergence. This Special Issue has contributions that address essential aspects related to its tolerance to the lack of oxygen. Publications that address rice aspects include reviews on the molecular regulatory pathways and the metabolic adaptation in the seed germination and early seedling growth [2,3]. A first review examines in detail aspects about the coleoptile elongation under submergence, anaerobic gene regulation in rice coleoptile, chromosomal regions regulating coleoptile elongation under oxygen shortage, starch degradation during anaerobic germination, and the hormonal regulation of anaerobic rice coleoptile elongation [2]. A second review focuses on the recent advances underlying anaerobic germination and coleoptile elongation and highlights the prospect of introducing quantitative trait loci (QTL) for anaerobic germination into rice mega varieties [3]. In addition, interesting experimental information—indicating that chlorophyll retention, content, low hydrogen peroxide accumulation, and catalase activity are related to better performance under submergence in seedlings of five japonica rice varieties—is also shown [4]. Conversely, another paper examined the potassium efflux and cytosol acidification as primary anoxia-induced events in wheat and rice seedlings and found that rice responses were more distinct and reversible upon reoxygenation when compared with sensitive wheat [5].

Root aeration is essential to withstand water excess scenarios such as waterlogging [6]. The formation of aerenchyma in roots is critical to enable the diffusive oxygen transport to reach the root tips. Additionally, this longitudinal transport of oxygen towards root tips can be constrained if there is an excessive radial loss of oxygen towards the rhizosphere (ROL). Hence, the presence of a barrier preventing ROL is a desirable trait for more efficient root aeration. In this regard, it is shown for rice that auxin-mediated signaling contributes to ethylene-dependent inducible aerenchyma formation in roots [7]. It was demonstrated that an auxin transport inhibitor stopped aerenchyma formation under oxygen-deficient conditions and reduced the expression of genes encoding ethylene biosynthesis enzymes [7]. Complementarily, another contribution assessed the formation of barrier to oxygen loss of four genotypes from two wild rice species (*Oriza glumaepatula* and *O. rufipogon*) and found that the three *O. glumaepatula* accessions formed a ROL barrier constitutively, while the accession of *O. rufipogon* accession did not [8]. Therefore, these wild relatives’ selected accessions might be crossed with elite commercial rice materials to incorporate or improve this root trait aiming at better root aeration when waterlogged [8].

The traits aiding to the recovery from submergence-induced hypoxia have been less examined and identified than those conferring tolerance during the stress period. A detailed study in the legume *Lotus japonicus* showed genotypic variation in the recovery ability (RGR) from short-term complete submergence and a trade-off between growth during vs. after the stress. In addition, an inverse relationship between growth after submergence and the shoot to root ratio (SR) was identified, where genotypes with low values of SR were able to maintain high stomatal conductance, a better leaf water status, and chlorophyll retention [9].

Among the consequences of flooded plants are the involvement of hormone and metabolic responses as well as enhanced production of reactive oxygen species (ROS). To achieve progress in the mechanisms underlying anoxia tolerance is crucial to develop a broader view considering interactions between different signaling pathways. An exciting overview of the hypoxia field’s classical and recent findings is reported in this Special Issue [10]. The review summarizes various aspects of low oxygen stress: (i) hypoxia sensing; (ii) adaptation to hypoxia at the cellular level; (iii) environmental hypoxia; and finally (iv) developmental hypoxia representing a physiologically relevant condition for the functionality of specific plant tissues [10].

The molecular mechanism of oxygen perception has been revealed in plants where proteins belonging to group VII Ethylene Response Factors (EFR-VIIs) play a pivotal role in becoming stable under hypoxia and degraded by proteasome machinery under aerobic conditions [11,12]. A new contribution provides information about the function of the *Jatropha curcas* ERFVIIs and the consequent N-terminal modification that stabilized the protein under low oxygen availability [13]. It was shown that JcERFVII2 is an N-end rule regulated waterlogging-responsive transcription factor that modulates gene expression under different stress-responsive conditions, including low-oxygen, oxidative, and pathogen response [13].

The involvement of hormones during hypoxia stress demonstrated that in root apical meristem, crosstalk between hypoxia and JA signaling occurs [14]. The jasmonate synthesis is initially enhanced but later decreased probably due to lack of oxygen and a consequent energy crisis [14]. Previous research has shown that when low oxygen occurs, ethylene signal drives hypoxia responses and improves survival in Arabidopsis [15,16]. A new paper showed that the hypoxia response triggered by ethylene is conserved in *Solanum* species, and, as it occurs in *Arabidopsis*, it enhances hypoxia tolerance [17]. One of ethylene signaling effects is hydrogen peroxide (H_2_O_2_) production that acts as a second messenger. To clarify the relationship between ethylene and H_2_O_2_ under low oxygen availability the rbohD/ein2-5 double mutant plants was analyzed under hypoxic stress [18]. The results demonstrated a synergistic interaction between ethylene and H_2_O_2_ signaling in modulating seed germination, seedling root growth, leaf chlorophyll content, and hypoxia-inducible gene expression [18].

In *Medicago truncatula*, the metabolic response differs between shoots and roots tissue [19]. Analyzing the composition of phloem exudate sap, they demonstrated that roots and leaves have distinct metabolic responses. Overall, the metabolomic data suggest that the decrease in sugar import in waterlogged roots increases the phloem sugar pool, which exerts a negative feedback regulation on sugar metabolism in shoots tissue [19].

In recent years, a group of non-coding RNA molecules, microRNAs (miRNAs), were identified. They play a pivotal role in different cellular processes. Among them, it was proposed to have a role in response to environmental stresses triggering the repression of target genes [20]. Taking advantage of high-throughput small RNA sequencing, a group of micoRNAs was identified in maize and teosinte under two crucial environmental stresses, submergence and drought and alternated stresses. Therefore, the identified miRNAs are a good starting point to establish the roles of miRNAs in stress response and could be useful for improving stress tolerance [21].

Finally, some practical aspects are also approached by articles on this Special Issue for other agricultural species. For instance, in *Physalis peruviana* (cape gooseberry), it was reported that foliar glycine betaine or hydrogen peroxide sprays ameliorate waterlogging stress, and that these can be potentially used as tools in managing waterlogging in this horticultural Andean fruit crop species [22]. Additionally, in *Pisum sativum* (pea) it was proved that the application of a hypoxic treatment decreases the physiological action of the herbicide imazamox due to an amelioration of the effects on total soluble sugars, starch accumulation, and changes in some amino acids [23]. This allows the authors to suggest that fermentation might constitute a plant defense mechanism that decreases the herbicidal effect [23]. Finally, a complete revision of the state of the art is provided regarding the findings that explain the traits conferring tolerance to root hypoxia in woody fruit species [24]. Special attention is given to the strategies for managing the energy crisis in *Prunus* species, and less explored topics in recovery and stress memory in woody fruit trees are pointed out.

## 3. Future Perspectives

The studies briefly summarized above provide an advance in our understanding of the molecular basis, the ecophysiological traits, and some of the genetic diversity of the model species *Arabidopsis thaliana*, *Lotus japonicus*, and *Medicago truncatula*. They also increase our understanding of some agricultural species (rice and its wild relatives, wheat, cape gooseberry, alfalfa, *Prunus* spp.) in response to waterlogging and submergence. These advances could provide opportunities to breed crops tolerant to oxygen deficiency. Despite the advances in knowledge gained over the last years, some challenges need to be addressed: (i) the study of genes indicative of ethylene-mediated hypoxia acclimation must be deepened, exploring the universality of discovered mechanisms of tolerance across species; (ii) physiological traits associated with plant recovery from submergence and their regulation must be identified; and (iii) trait-to-gene-to-field approaches (i.e., “translational research”), warranting the development of strategies to cope with oxygen deficiency stress aiming to stabilize crop yields, must be promoted to resolve food insecurity in the future. The coordinated effort of research groups working at the different organization levels (e.g., molecular, plant and field) will increase the chances of success of the “translational research” as the main goal, implying the translation of basic scientific discovery into improved agricultural productivity.
